# Assessing the predictive validity of Provincial Medical & Dental College Admission Test (MDCAT)

**DOI:** 10.12669/pjms.40.5.7766

**Published:** 2024

**Authors:** Saira Rahim, Mahwish Arooj

**Affiliations:** 1 Saira Rahim Director of Medical Education, Pak Red Crescent Medical and Dental College, Lahore, Pakistan; 2 Mahwish Arooj The Principal, University College of Medicine and Dentistry University of Lahore, Pakistan

**Keywords:** Entry exam, Medical admission, Performance trajectory, Summative exams

## Abstract

**Objective:**

To assess the predictive validity of Punjab Provincial Medical & Dental College Admission Test (MDCAT).

**Methods:**

A retrospective correlational study was conducted at the Fatima Memorial Hospital College of Medicine & Dentistry in Lahore, Pakistan. Data were analysed from 415 medical students who had completed the MBBS program from 2018-2020. Bivariate and multivariable regression models were used to adjust for confounders such as age, gender, city of origin, and pre-medical performance. A novel performance-trajectory analysis was used to evaluate whether students with different preadmission MDCAT scores had different performance trajectories in medical school.

**Results:**

On simple bivariate analysis, there was a weak but significant (correlational coefficient 0.22-0.33, p<0.001) correlation between MDCAT scores and professional exam scores for all years of medical college. However, multivariable analysis of Matric/Fsc track showed no significant correlation (p > 0.05) between MDCAT and professional exam scores after the first two years. For O/A level students, there was no correlation between MDCAT and professional exam scores for any year of medical college (p>0.05). Trajectory analysis showed that students with exceptionally high MDCAT scores had a superior performance trajectory compared to those with the lowest quartile of MDCAT scores while students with average MDCAT scores had overlapping performance trajectories.

**Conclusion:**

For students from the Matric/FSc track only, there is a weak but significant correlation between MDCAT scores and professional exam scores for the first two years. No such correlation exists for O/A level students. Exceptionally high MDCAT scores are predictive of higher achievement in professional exam scores.

## INTRODUCTION

In Pakistan, Medical and Dental College Admission Test (MDCAT) is utilized for student selection in medical colleges. Such emphasis is placed on the importance of this exam in admissions that mental health ramifications on prospective applicants have been documented^1^ and multiple stakeholders have voiced concerns about perceived fairness.^2^ The problem motivating the present study was that the exam has not been robustly validated in terms of its correlation with long-term performance of students from Matric/FSc and O/A level tracks separately. This is a major gap in the existing literature for multiple reasons. Firstly, we believe it is desirable to document in the academic literature whether or not the longitudinal data support the notion that MDCAT scores and medical student performance are correlated specifically within the Pakistani medical education system. Secondly, we find that the existing literature has largely ignored the fact that students from Matric/FSc and O/A Level tracks are currently being evaluated using a common examination without any subgroup analysis supporting this methodology; this is problematic given that prior studies have shown that premedical school system is an important factor in medical school performance in Pakistan.^3^

Our objectives in this study are: 1) to evaluate whether MDCAT entry scores can be validated in terms of student performance in medical college, 2) to identify whether the validity of these scores varies as function of pre-admission school system (i.e. Matric/FSc and O/A Level tracks). The significance of our work is that failure to validate the correlation of MDCAT scores in terms of student performance may constitute grounds for re-evaluation of the significant weightage that MDCAT scores are currently afforded in premedical admissions in Pakistan.

## METHODS

The study analysis was conducted from June 2021 to June 2022 using data from 2013 to 2020. The study was designed as a retrospective cohort study using various quantitative methods as described in detail below.

The study sample consisted of medical students of Fatima Memorial Hospital College of Medicine & Dentistry (FMHCMD), Lahore, a major academic centre in Lahore, Pakistan. The initial data capture yielded 450 students who were enrolled in the MBBS program in 2013, 2014, and 2015 and thus graduated from the five-year curriculum in 2018, 2019, and 2020. There were no significant changes in the format or content of the MDCAT examination in these three years. Students who failed to graduate within their allotted five-year window were excluded from the study. After applying these inclusion and exclusion criteria, the final sample consisted of 415 students.

### Ethical Approval:

This study was conducted after approval from the FMH Institutional Review Board dated September 23, 2021, Reference Number FMH-07-2021-IRB-931-M. The study subsequently also underwent full review and approval by the Ethical Review Board of the University of Lahore (Reg. No. ERC/83/21/12.

Data retrieval required manual review of student records for each student. Data were compiled into a single Microsoft Excel spreadsheet. No student identifying data elements were included in the dataset. Demographic characteristics for each student such as age, gender, and city of origin were included. Pre-admission scores including MDCAT and either Matric/FSc scores or O/A equivalence scores were also included, as well as scores for first to final year medical school Professional examination.

For inferential analysis, linear regression was used to model student Professional exam scores for each year of medical school as the outcome variable. The predictor variable of interest was the MDCAT score for each student. Initially, to investigate whether there was a simple unadjusted correlation between pre-admission MDCAT scores and the summative Professional exam scores of each year of medical school, bivariate linear regression was employed. Thereafter, multivariable linear regressions were built. In the latter, multiple confounding variables were adjusted for including demographics and pre-medical school performance scores. The multivariable linear regression models also included interaction terms. Interaction terms are a feature of regression modelling which allows the model to account for the fact that two or more variables may have a significant interaction i.e. be correlated or linked in some way.

For performance trajectory analysis, students were stratified into four distinct groups based on their performance in the MDCAT. The overall sample was divided into four quartiles, Quartile one included the students with the lowest MDCAT scores while Quartile four included the students with the highest MDCAT scores. The average summative Professional exam performance for all Professional exams of each MDCAT strata was plotted over time on a shared set of axes to yield four different ‘performance trajectories’ based on initial MDCAT scores. Demonstration that the regression model faithfully represents the dataset was accomplished by showing actual-by-predicted scatterplots and citing the associated Pearson correlation coefficient for the model as well as the associate p values (p<0.05 was considered significant.) Statistical analysis was conducted using SPSS version 25 statistical package. Performance trajectory analysis was conducted using Microsoft Excel.

## RESULTS

Key descriptive statistics to describe the student population are sown in Table-I. A majority of the sample consists of female students (65%). In general, students were of a similar age at the time of graduation as demonstrated by a relatively narrow standard distribution (1.7 years) with an average age of 26.2 years. Notably, over half of students (55%) were geographical locals (i.e. from the city of Lahore.) An overwhelming majority of students had arrived in the medical school through the Matric/FSc premedical pathway (80%) while only a minority completed the O/A Level pathway (20%).

**Table-I:** Key Descriptive Statistics of Study Student Population.

Gender (N = 415)	*Male (%)*	*Female (%)*
	35	65
Average Age in Years (Standard Deviation) (N = 415)	26.4 (1.7)	
Residents of Lahore (%) (N = 415)	55	
Pre-medical School System (N = 415)	*O/A Level %*	*Matric/FSC %*
	20	80
Matric Score % (Standard Deviation) (N= 328)	88 (7.4)	
O Level Equivalence Score % (Standard Deviation) (N = 82)	82 (6.4)	
FSc Score % (Standard Deviation) (N = 338)	85 (5.2)	
A Level Equivalence Score % (Standard Deviation) (N = 75)	81 (6.5)	
MDCAT Score % (Standard Deviation) (N = 400)	76 (11)	
Average Prof 1 % (Standard Deviation) (N = 412)	67 (5.5)	
Average Prof 2 % (Standard Deviation) (N = 413)	71 (6.4)	
Average Prof 3 % (Standard Deviation) (N = 412)	71 (6.3)	
Average Prof 4 % (Standard Deviation) (N = 412)	69 (6.3)	
Average Final Prof % (Standard Deviation) (N = 412)	72 (4.6)	

Results from bivariate analysis are summarized in Table-II. In brief, simple bivariate linear regression shows that a weak (coefficients < 0.5) but statistically significant (p<0.05) positive correlation exists between MDCAT scores and Prof scores for each year of medical school but the correlation becomes progressively weaker over the course of a student’s medical school career.

**Table-II:** Results of linear regression modelling to find correlation between MDCAT and Prof Scores.

	Bivariate or Multivariate	Matric/FSC students or O/A level students	Prof 1 Score %	Prof 2 Score %	Prof 3 Score %	Prof 4 Score %	Prof 5 Score %	Longitudinal correlation present?
Correlation coefficient with MDCAT Score	Bivariate	Combined	0.33	0.38	0.29	0.26	0.22	Weak but significant correlation between MDCAT scores and summative scores for all years of medical school on bivariate modelling
p value of correlation coefficient	Bivariate	Combined	<0.001	<0.001	<0.001	<0.001	<0.001	
Beta-coefficient (bivariate linear regression model)	Bivariate	Combined	0.17	0.22	0.18	0.16	.10	
p value of beta-coefficient	Bivariate	Combined	<0.001	<0.001	<0.001	<0.001	<0.001	
R^2^value for overall model	Multivariate	Matric/FSC	0.43	0.37	0.33	0.37	0.44	NA
p value of overall model	Multivariate	Matric/FSC	<0.001	0.0006	0.02	0.001	<0.001	
Parameter estimate for MDCAT Score %	Multivariate	Matric/FSC	0.19	0.26	0.16	0.15	0.09	Weak but significant correlation for first 2 years, no significant correlation thereafter
p value for MDCAT Score % variable	Multivariate	Matric/FSC	0.006	0.002	0.06	0.08	0.15	
R^2^value for overall model	Multivariate	O/A Level	0.38	0.36	0.40	0.41	0.6	NA
p value of overall model	Multivariate	O/A Level	0.006	0.01	0.0049	0.001	<0.001	
Parameter estimate for MDCAT Score %	Multivariate	O/A Level	0.17	0.19	0.02	0.08	-0.02	No statistically significant correlation between MDCAT scores and summative scores at any time.
p value for MDCAT Score % variable	Multivariate	O/A Level	0.1	0.1	0.8	0.4	0.7	

While bivariate analysis suggests that a correlation may exist between MDCAT scores and medical school performance, such an analysis does not take into account differences in the baseline characteristics (demographics and pre-admission academic performance) of students. In order to account for these differences, multivariable linear regression models were used. Because students were admitted from two separate school systems, separate models were built for students from the Matric/FSc pathway and the O/A Level pathway. The parameter estimates for the correlation between MDCAT scores and Prof scores for each year of medical school for Matric/FSc students are summarized in Table-II.

The table shows that there is a very weak but statistically significant correlation between MDCAT scores and Professional scores for the first two years of medical school only. The correlation is considered weak due to correlation coefficients in the 0.2-0.3 range even though they are statistically significant (p<0.05). Thereafter, no statistically significant correlation between MDCAT scores and Professional scores is discernable. Thus, the correlation between MDCAT score and Professional scores that was noted in the simple bivariate analysis was not noted in the multivariable analysis after adjusting for baseline differences in demographics and pre-admission academic performance.

Table-II also summarizes the parameter estimates for the correlation between MDCAT scores and Professional scores for each year of medical school for O/A level students. The table shows that there is no statistically significant correlation between MDCAT scores and Professional scores for any year of medical school in the case of O/A level students.

To conduct trajectory analysis, students were divided into four quartiles on the basis of pre-admission MDCAT score. Each quartile contained a quarter of the student group. Students in Quartile-1 had the lowest MDCAT scores while students in Quartile four had the highest MDCAT scores. Performance trajectories are shown based on the average Professional scores for each year of the MBBS curriculum.

**Fig.1:**
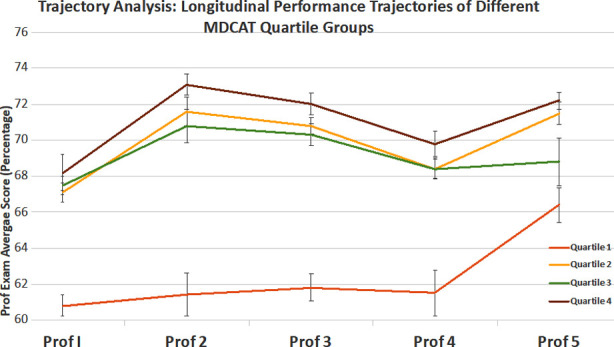
Trajectory analysis: longitudinal performance trajectories of different MDCAT quartile groups.

The trajectory analysis (Fig.1) shows that students from Quartile one and Quartile four have markedly different performance trajectories, based on average Professional scores. However, students from the middle two quartiles i.e., Quartile two and Quartile three have overlapping trajectories over the course of medical school. The analysis suggests that medical school performance trajectories may correlate with pre-admission MDCAT scores for Quartile four students who had extremely high scores and for Quartile 1 who had lowest scores.

## DISCUSSION

When a multivariable regression was applied which adjusted for baseline differences in demographics and pre-admission academic performance metrics, we noted that for Matric/FSc track students very weak correlation existed between MDCAT scores and Professional scores for the first two years of medical school. Thereafter, there was no statistically significant correlation between MDCAT scores and Professional scores. Crucially, there was no statistically significant correlation between MDCAT scores and Professional scores for O/A level students at any point in their medical school career. Thus, our study is the first to show that the correlation of MDCAT scores and Professional scores varies as a function of pre-medical school system in Pakistan.

It is worthwhile to discuss our findings in light of the existing Pakistani literature on this topic. In contrast to other notable studies which only studied correlation between pre-admission tests and student performance in the first or second year of medical school,^4^-^9^ the present study provides insight into the longitudinal correlation or lack thereof between MDCAT scores and medical school performance. Many of these earlier studies suggested that a weak correlation existed between MDCAT and early Professional exam performance; our work recapitulates this finding but adds the important nuance that the correlation is very short-lived and cannot be demonstrated after the second year of medical school. Furthermore, our study shows that the correlation only exists for students from the Matric/FSc pathway and is not demonstrable for O/A level students.

It is also worthwhile to see our results in the context of the international medical education literature. In an early study from the American Association of American Medical Colleges (AAMC), Jones et al provided evidence that there was a correlation between MCAT scores and medical school performance in the first two years^10^ a result that we have seen demonstrated in our own analyses. Dixon et al have previously shown that a correlation existed between MCAT scores and performance in both COMPLEX-USA Level-1 and Level-2.^11^ Likewise, Gauer et al have reported a weak but significant correlation between MCAT scores and USMLE Step-1 and Step-2 CK scores.^12^

Our work has added the following important findings to the existing literature: 1) we provide evidence that MDCAT scores do not have long term correlation with medical school performance, and their weightage in medical school admission criteria should thus be re-evaluated accordingly, 2) we are the first to show that the predictive capacity of MDCAT varies as a function of pre-medical school system (i.e. Matric/FSc vs O/A Level), 3) Our study is the first to employ trajectory-based analysis to the study of medical student performance globally. The especially compelling result of our trajectory-based analysis is that students with exceptionally high or low MDCAT scores had different performance trajectories in medical school but those with average score i.e., the middle two quartiles were not distinguishable in terms of performance trajectories.

### Limitations:

The study is a retrospective correlational analysis. As with any retrospective study, one of the main limitations is that investigators were only able to include variables which could be accessed retrospectively in the analysis such as age and gender. The existing literature has previously shown that medical school performance is impacted by many other important variables including parental income^13^,^14^ and racial or ethnic minority status^15^,^16^ These variables were not available for retrospective collection in the present study.

Our study is based on data from a single large academic institution in a major urban centre. Though the analyses are meaningful in their own right, they may not represent the results of students throughout the country. Previous studies have suggested that a rural versus urban divide persists in the world of medical education^17^ and so the results of studies from major urban centres may not be representative of the rural situation. Future studies would be well placed to adopt a multi-institutional data-sharing model to overcome this limitation.

## CONCLUSION

The MDCAT has a weak but significant correlation with medical student performance for the first two years of medical school for students who entered medical training from the Matric/FSc pathway. No such correlation exists for students who entered medical training through the O/A level pathway. The unadjusted performance trajectories of medical students stratified based on pre-admission MDCAT scores show that students who scored exceptionally highly in the MDCAT are likely to have different performance trajectories compared to the lowest scoring students. However, students with average MDCAT scores (middle two quartiles) cannot reliably be distinguished based on performance trajectories.

### Recommendation:

Future studies on the role of pre-admission standardized testing in Pakistan should be prospective, multi-institutional, and should include additional variables such as household income and minority group status. The overall landscape of Pakistani medical school admissions is likely to shift, in accordance with international trends, towards a more holistic evaluation of applicant quality which includes use of extra-curricular portfolios, letters of recommendation, and multiple mini-interviews.^18^,^19^
